# Alterations in pathogen-specific cellular and humoral immunity associated with acute peripheral facial palsy of infectious origin

**DOI:** 10.1186/s12974-023-02933-4

**Published:** 2023-10-25

**Authors:** Leyla Mohammad, Mathias Fousse, Gentiana Wenzel, Marina Flotats Bastardas, Klaus Faßbender, Ulrich Dillmann, Bernhard Schick, Michael Zemlin, Barbara C. Gärtner, Urban Sester, David Schub, Tina Schmidt, Martina Sester

**Affiliations:** 1 Department of Transplant and Infection Immunology, Saarland University, 66421 Homburg, Germany; 2 Department of Neurology, Saarland University, Homburg, Germany; 3 Department of Otorhinolaryngology, Saarland University, Homburg, Germany; 4 Department of Pediatrics and Neonatology, Saarland University, Homburg, Germany; 5 Department of Medical Microbiology and Hygiene, Saarland University, Homburg, Germany; 6 SHG Klinikum Völkingen, Völklingen, Germany

**Keywords:** Peripheral facial palsy, T cells, Cellular immunity, VZV, HSV, Borrelia

## Abstract

**Background:**

Peripheral facial palsy (PFP) is a common neurologic symptom which can be triggered by pathogens, autoimmunity, trauma, tumors, cholesteatoma or further local conditions disturbing the peripheral section of the nerve. In general, its cause is often difficult to identify, remaining unknown in over two thirds of cases. As we have previously shown that the quantity and quality of pathogen-specific T cells change during active infections, we hypothesized that such changes may also help to identify the causative pathogen in PFPs of unknown origin.

**Methods:**

In this observational study, pathogen-specific T cells were quantified in blood samples of 55 patients with PFP and 23 healthy controls after stimulation with antigens from varicella-zoster virus (VZV), herpes-simplex viruses (HSV) or borrelia. T cells were further characterized by expression of the inhibitory surface molecule CTLA-4, as well as markers for differentiation (CD27) and proliferation (Ki67). Pathogen-specific antibody responses were analyzed using ELISA. Results were compared with conventional diagnostics.

**Results:**

Patients with PFP were more often HSV-seropositive than controls (*p* = 0.0003), whereas VZV- and borrelia-specific antibodies did not differ between groups. Although the quantity and general phenotypical characteristics of antigen-specific T cells did not differ either, expression of CTLA-4 and Ki67 was highly increased in VZV-specific T cells of 9 PFP patients, of which 5 showed typical signs of cutaneous zoster. In the remaining 4 patients, a causal relationship with VZV was possible but remained unclear by clinical standard diagnostics. A similar CTLA-4- and Ki67-expression profile of borrelia-specific T cells was also found in a patient with acute neuroborreliosis.

**Discussion:**

In conclusion, the high prevalence of HSV-seropositivity among PFP-patients may indicate an underestimation of HSV-involvement in PFP, even though HSV-specific T cell characteristics seem insufficient to identify HSV as a causative agent. In contrast, striking alterations in VZV- and borrelia-specific T cell phenotype and function may allow identification of VZV- and borrelia-triggered PFPs. If confirmed in larger studies, antigen-specific immune-phenotyping may have the potential to improve specificity of the clinical diagnosis.

**Supplementary Information:**

The online version contains supplementary material available at 10.1186/s12974-023-02933-4.

## Background

Peripheral facial palsy (PFP) is a common neurological symptom consisting of an incomplete or complete loss of signal transmission by the facial nerve resulting in a variable degree of mainly unilateral palsy of mimic muscles. The severity of symptoms is indicated by House–Brackmann grading [1]. Approximately 60–75% of patients are diagnosed with idiopathic PFPs of unknown origin, also termed Bell’s palsy [2]. Herpes-simplex virus (HSV) has been suggested to be causally related to idiopathic PFPs to some extent, although its involvement is still controversially discussed [3, 4]. In general, non-idiopathic PFPs are often triggered by infectious pathogens, with most frequent involvement of varicella-zoster virus (VZV; with or without simultaneous skin disease), or borrelia (neuroborreliosis) [5–8]. In addition, although less frequent, administration of vaccines, or autoimmune or neoplastic diseases can also be causally linked to acute PFPs. Moreover, trauma, tumors, cholesteatoma and further local conditions disturbing the function of the peripheral part of the nerve have to be ruled out [9, 10].

The choice of a specific therapeutic regimen is difficult. As a consequence, based on provisional evaluation during the first visit, PFPs are treated with steroids, anti-viral and/or anti-bacterial agents, although an inappropriate treatment regimen may bear the risk of prolonged symptoms or permanent damage of the nerve [11, 12]. A combination therapy is recommended in unclear cases or when pathogen diagnosis is delayed [13, 14]. A faster and more specific diagnosis of the causative agent of PFP would facilitate a specific choice of therapy.

Cerebral imaging may be considered to detect neoplastic processes or brainstem lesions [15]. Electrophysiological procedures provide evidence of early hypoexcitability in the facial canal (typical in idiopathic PFP), but this is not specific for the etiology of PFP [16]. Analysis of cerebrospinal fluid (CSF) is useful for determination of pleocytosis, of pathogenic nucleic acids, and/or of intrathecal increase of pathogen-specific antibodies compared to blood (antibody specific index, ASI) [17, 18]. However, a lumbar puncture is sometimes not possible due to limited compliance, anatomical abnormalities or coagulations disorders [19]. Detection of pathogen-specific nucleic acids and IgA or IgM from blood samples is less invasive, but the diagnostic window for nucleic acid detection in blood is small, and specificity of antibodies is poor. Therefore, identification of the underlying cause of PFP is difficult and results in non-specific or symptomatic treatment regimens.

Analysis of pathogen-specific T cell responses has shown that effective pathogen control is not only determined by the quantity of pathogen-specific T cells, but also by their functional and phenotypic properties. Active infection with cytomegalovirus (CMV) after solid organ transplantation was associated with an increase in the expression of the inhibitory surface molecule PD-1 on CMV-specific T cells [20]. Likewise, VZV-specific T cells during active herpes zoster show phenotypic alterations characterized by an increased expression of CTLA-4 and PD-1 [21]. These VZV-specific T cell characteristics were found in both patients with skin rashes and with VZV-related central nervous system (CNS) infections [22]. Thus, an increased CTLA-4 expression may serve as a highly sensitive marker for identification of VZV-related CNS-infections, even in the absence of a VZV-rash [22]. We therefore hypothesized that alterations in pathogen-specific T cells may be used to identify the causative pathogen in patients with PFPs.

## Materials and methods

### Recruitment of the study population

In an observational study, patients with clinically diagnosed acute PFP were recruited at the Department of Neurology, Department of Otorhinolaryngology and Department of Pediatrics and Neonatology of Saarland University between September 2017 and February 2019. The number of study participants was determined by feasibility of analyses and recruitment, and no exclusion criteria were applied. Twenty-three age-matched healthy individuals served as control group. Clinical grading of PFP was determined using the House–Brackmann score [1]. All patients received standard routine diagnostics including determination of pleocytosis and analysis of pathogens by PCR or microscopy in CSF, electrodiagnostic testing, assessment of response to treatment, and/or screening for VZV- or borrelia-related skin manifestation. Diagnostic parameters and treatment were chosen by the treating physicians and decisions were unaffected by results of the current study. To address clinical grading and stability of immunological parameters during follow up, a subgroup of PFP-patients and controls who volunteered to provide blood samples were analyzed approximately two weeks after the first sample acquisition.

### Quantitative and functional analysis of pathogen-specific T cells

Analysis of pathogen-specific T cell responses in whole blood samples was performed as described before [21, 23]. In brief, heparinized whole blood was stimulated with VZV-lysate, HSV-1/-2-lysate, CMV-lysate (32 µl/ml each; Virion/Serion) and a mix of bulk antigens from *Borrelia garinii* and *Borrelia afzelii* (10 µg/ml each; Virion/Serion), respectively. Stimulations with uninfected control lysates (32 µl/ml each; Virion/Serion) and 2.5 µg/ml *Staphylococcus aureus* enterotoxin B (SEB; Sigma), respectively, served as negative and positive control. All stimulations were carried out in the presence of 1 µg/ml anti-CD28 and anti-CD49d costimulatory antibodies (BD Biosciences). After 2 h, brefeldin A was added for intracellular accumulation of induced cytokines. After additional 4 h, cells were EDTA-treated and fixed as described before [21, 23].

Two separate staining reactions were performed to analyze surface markers (CD4, CD27, CD69, CTLA-4) and intracellular molecules (IFNγ, Ki67) after permeabilization of cells with a saponin-containing buffer (BD Biosciences). Intranuclear expression of Ki67 was detected using the “Foxp3/Transcription Factor Staining Buffer Set” (ThermoFisher) in combination with anti-Ki67 antibodies (BD Biosciences). 300 µl of whole blood were used per stimulatory reaction and staining. If blood volume was limited, staining for CD27 and Ki67 was omitted. At least 10,000 CD4 T cells per sample were flow-cytometrically analyzed using a BD FACS Canto II and the BD FACSDiva software version 6.1.3.

### Semi-quantitative and quantitative analysis of pathogen-specific antibodies

VZV-, HSV- and CMV-specific IgG-levels in blood were quantified using anti-IgG enzyme-linked immunosorbent assays (ELISA, Euroimmun, Lübeck, Germany). Ratios of VZV- and HSV-specific IgAs and HSV-specific IgMs were semi-quantitatively determined by anti-IgA and -IgM ELISA (Euroimmun), respectively. Borrelia-specific IgGs and IgMs were screened using the chemiluminescence immunoassay (CLIA) technology (Liaison^®^, DiaSorin). Intermediate and positive screening results were subsequently confirmed by line-immunoblot (Borrelia ViraStripe®, Viramed). Cut-offs to define negative, intermediate and positive responses were defined as per manufacturer’s instructions.

### Statistical analysis

Statistical analysis was performed using GraphPad-Prism 10.0.0. Quantitative non-normally distributed variables between controls and PFP-patients were compared using Mann–Whitney test. Analysis of quantitative parametric values (age, time between 1st and 2nd blood sampling) was performed using the unpaired t-test. The Wilcoxon matched pairs test was performed to compare quantitative T cell responses over time. Differences in sex distribution or serostatus between groups were analyzed by Fisher’s exact test. Cut-off values for VZV-, HSV- and CMV-specific CD4 T cells (0.02%, 0.025% and 0.05%, respectively) were calculated by ROC-analysis of T cell responses in seronegative and seropositive healthy individuals and/or as defined before [21, 24]. As borrelia-specific antibody responses are of limited value, calculation of a cut-off for borrelia-specific T cells based on serology was not considered reasonable. Therefore, the lowest cut-off of 0.02% was chosen for borrelia-specific T cells in this study.

## Results

### Study population

Fifty-five patients with PFP and 23 healthy controls were recruited. Demographic and clinical characteristics of the study population are summarized in Table 1. Patients showed higher leukocyte counts (*p* = 0.042), and their percentage of lymphocytes was significantly lower (*p* = 0.002). The neutrophil/lymphocyte ratio as a prognostic hematologic marker of Bell's palsy [25] was also significantly higher in patients than in controls (*p* = 0.004). The intensity of PFP indicated by the House–Brackmann grading was moderate to high. The treatment regimens were based on results of standard clinical parameters and procedures (Table 1).

**Table 1:** Characteristics of the study populations

	PFP-patients	Controls	p-value
*n*	55	23	
Years of age [mean ± SD]	46.89 ± 19.3	43.1 ± 17.4	0.413
Females [n (%)]	21 (38.2%)	14 (60.9%)	0.089
Leukocytes/µl blood [median (IQR)]	7200 (3260)	6980 (2370)	0.042
% lymphocytes in blood [median (IQR)]	26.0 (11.1)	33.1 (5.4)	0.002
Neutrophil/lymphocyte ratio [median (IQR)]	2.42 (1.47)	1.73 (0.48)	0.004
Days between 1st and 2nd blood sampling, [mean ± SD]	13.8 ± 2.1 (*n = *36)	14.0 ± 1.0 (*n = *15)	0.996
Clinical characteristics PFP			
Days since onset of symptoms [mean ± SD]	2.6 ± 2.1	n.a	
House–Brackmann grading (1–6) [median (IQR)]^#^	3.5 (1.5)	n.a	
*PFP side*			
Left Right Bilateral	32 (58.2%) 21 (38.2%) 2 (3.6%)	n.a n.a n.a	
*Lumbar puncture*^§^	(*n = *47)	n.a	
Normal cell counts (0–4 cells/µl) Borderline (5 cells/µl) Pleocytosis (> 5 cells/µl)*	35 (74.5%) 4 (8.5%) 8 (17.0%)	n.a n.a n.a	
*PFP-treatment [n]*^$^	52	n.a	
Steroids	32		
Anti-bacterial^b^	1		
Steroids/anti-viral^a^	2		
Anti-viral^a^/anti-bacterial^b^	7		
Steroids/anti-viral^a^/anti-bacterial^b^	8		
Immunoglobulins	2		

^#^House–Brackmann grading was not available in 3 patients; 1–6 corresponds to I–VI

^$^3 patients with symptomatic treatment only

^§^lumbar puncture was not possible in 8 cases due to either oral anticoagulation (*n = *6) or lack of consent (*n = *2)

*median 14 (IQR 70.8) cells/µl

^a^i.e., (val)acyclovir

^b^i.e., ceftriaxone, doxycycline or ampicillin

### Correlation of pathogen-specific T cell frequencies and antibody levels in patients and controls

As VZV, HSV and borrelia are among the main causes of infectious PFP, specific humoral and cellular immune responses against these pathogens were analyzed. In addition, CMV-specific immune responses were assessed as a control, as its involvement with PFP is rare. The serostatus including IgG-, IgA- (VZV and HSV) and IgM-levels (HSV and borrelia) is shown in Table 2. Most individuals were positive for VZV-IgG, whereas positive or intermediate VZV-IgA levels were found in 43.6% of PFP-patients and 21.7% of controls (*p* = 0.078, Table 2). The percentage of HSV-IgG-positive individuals was significantly higher in PFP-patients (90.9%) than in controls (52.2%, *p* = 0.0003). Regarding other HSV Ig-classes, 47.3% of the tested individuals were IgA-positive, and 3.6% were IgM-positive with no difference between the groups (*p* = 0.133 and *p* = 0.470, respectively). The percentages of individuals with borrelia-IgG and IgM was low in both groups, with only one patient having both borrelia-IgG and IgM. In line with German seroprevalence, about 50% of all individuals were CMV IgG-positive.

**Table 2:** Serostatus of the study population

n (%)			Immunoglobulin levels			*p*-value*
			Negative	Intermediate	Positive	
VZV	IgG	Controls	0 (0)	0 (0)	23 (100)	p = 0.551
		PFP	3 (5.5)	1 (1.8)	51 (92.7)	
	IgA	Controls	18 (78.3)	3 (13.0)	2 (8.7)	p = 0.078
		PFP	31 (56.4)	2 (3.6)	22 (40.0)	
HSV	IgG	Controls	11 (47.8)	0 (0)	12 (52.2)	p = 0.0003
		PFP	5 (9.1)	0 (0)	50 (90.9)	
	IgA	Controls	13 (56.5)	2 (8.7)	8 (34.8)	p = 0.133
		PFP	20 (36.4)	9 (16.4)	26 (47.3)	
	IgM	Controls	19 (82.6)	3 (13.0)	1 (4.3)	p = 0.470
		PFP	49 (89.1)	4 (7.3)	2 (3.6)	
Borrelia	IgG	Controls	19 (82.6)	2 (8.7)	2 (8.7)	p > 0.9999
		PFP	47 (85.5)	2 (3.6)	6 (10.9)	
	IgM	Controls	23 (100)	0 (0)	0 (0)	p > 0.9999
		PFP	53 (96.4)	0 (0)	2 (3.6)	
CMV	IgG	Controls	12 (52.2)	0 (0)	11 (47.8)	p = 0.626
		PFP	25 (45.5)	0 (0)	30 (54.5)	

^*^p-values were calculated by Fisher’s exact test, where patients with intermediate and positive results were compared with negative results

**Fig. 1:**
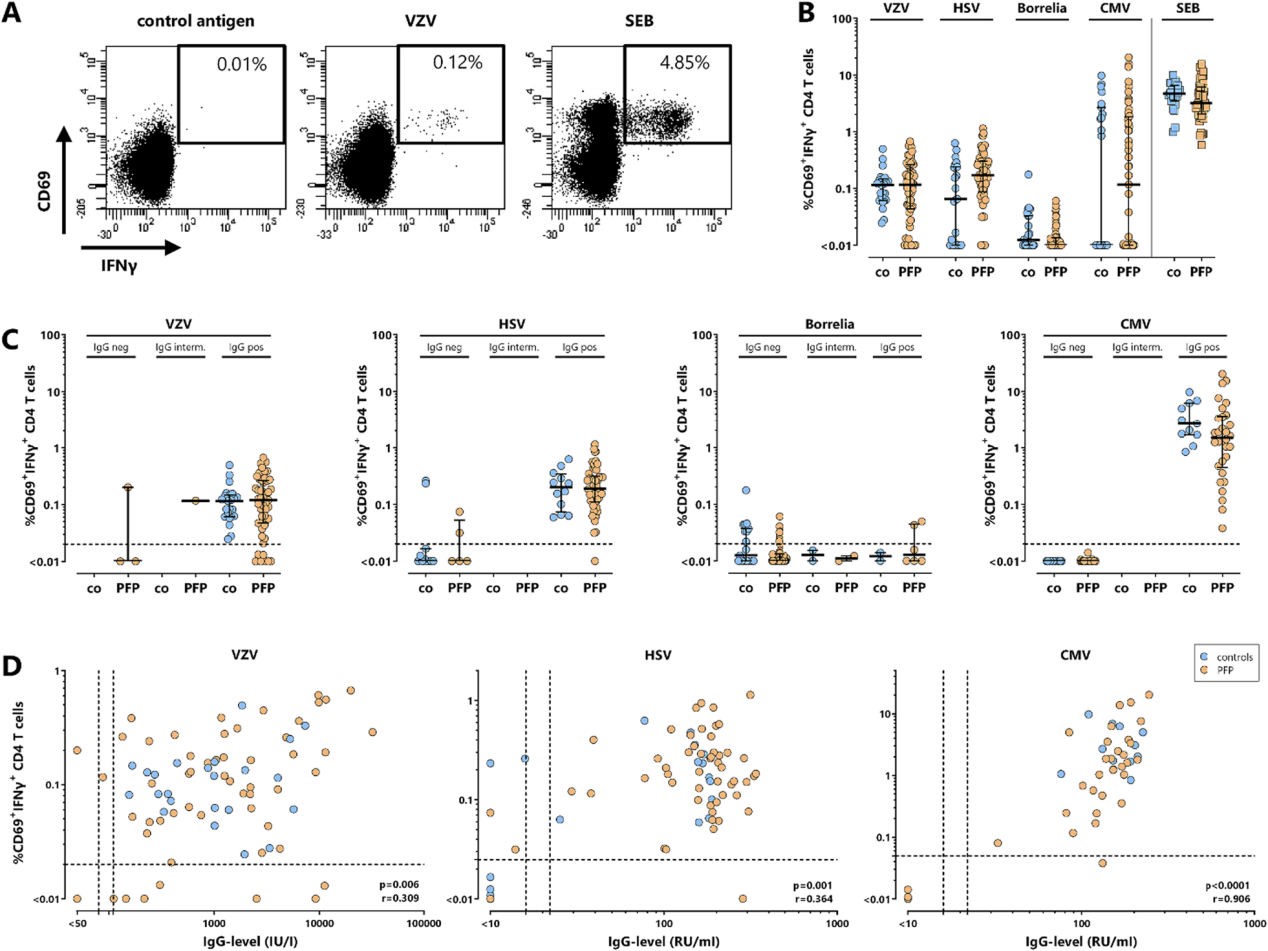
Correlation of pathogen-specific T cells and antibodies in patients with acute PFP and healthy controls. **A** Representative dot plots of CD4 T cells of a 36-year-old healthy control are shown after stimulation with either control lysate (control), VZV-lysate (VZV) or polyclonal stimulus *Staphylococcus aureus* enterotoxin B (SEB, positive control). Numbers indicate percentages of reactive CD4 T cells of total CD4 T cells and are characterized by co-expression of the activation marker CD69 and the cytokine IFNγ. In **B** percentages of VZV-, HSV-, borrelia-, CMV-specific and SEB-reactive CD4 T cells were compared between 23 controls (co, blue circles) and 55 patients with PFP (orange circles). **C** Percentages of pathogen-specific T cells in controls and PFP-patients were stratified according to the corresponding IgG-serostatus, whereas in **D** these percentages were correlated with corresponding IgG-levels (VZV, HSV, CMV). As determination of borrelia-specific antibodies relied on a two-step screening and confirmation system with semi-quantitative output, analysis of borrelia-specific IgGs was restricted to semi-quantitative analysis. In panel B and C median values are indicated for each group. There are no significant differences between controls and PFP-patients in panels B and C. Dotted lines in panel C and D represent detection limits for pathogen-specific CD4 T cells or IgG-levels. CMV, cytomegalovirus, HSV, herpes-simplex viruses; IFN, interferon; PFP, peripheral facial palsy; VZV, varicella-zoster virus

To quantify pathogen-specific T cells, whole blood samples of controls and PFP-patients were stimulated with pathogen-derived antigens in vitro. Stimulation with SEB was carried out to quantify polyclonal T cell responses. Representative dot plots of CD4 T cells of a 36-year-old healthy control after stimulation with negative control antigen, VZV-antigen and SEB are shown in Fig. 1A. Median percentages of pathogen-specific T cells did not differ between PFP-patients and controls, independent of their serostatus (Fig. 1B and C, all *p* > 0.05). Except for borrelia-specific immunity, T cell frequencies were generally highest for IgG-seropositive individuals, and the percentage of pathogen-specific T cells showed a significant correlation with corresponding IgG-levels (Fig. 1D).

### No distinct differences in phenotypical and functional parameters of pathogen-specific T cell responses between patients and controls

**Fig. 2:**
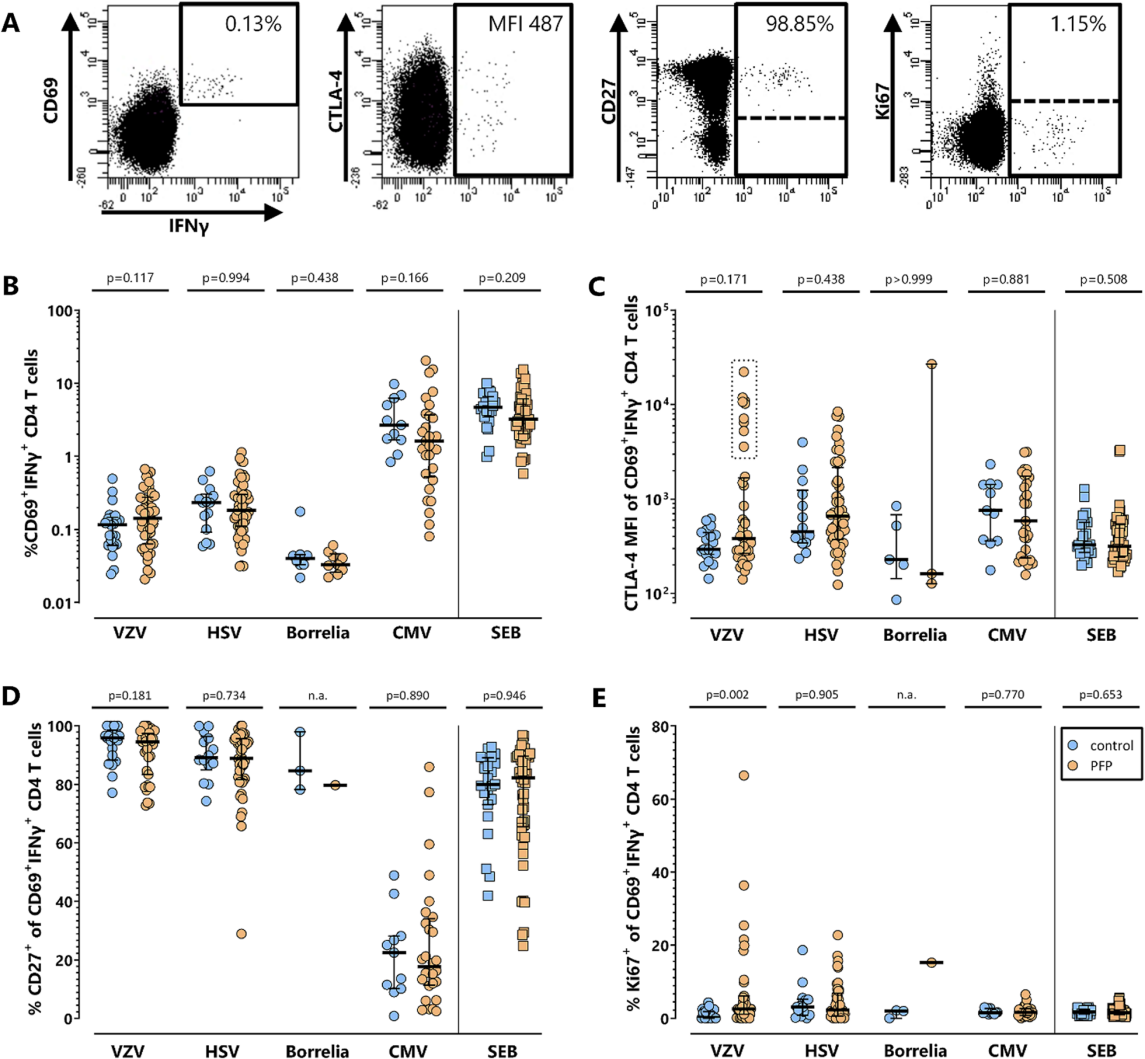
No distinct alterations in phenotype and proliferation of pathogen-specific T cells in patients with PFP. T cell responses in controls and PFP-patients with detectable pathogen-specific T cell frequencies were quantitatively and qualitatively characterized further. In **A** representative dot plots of VZV-stimulated CD4 T cells of a 28-year-old PFP-patient are shown. Percentages of pathogen-specific CD4 T cells (**B**), their expression of CTLA-4 (**C**), and the percentage of pathogen-specific cells positive for CD27 (**D**) or Ki67 (**E**) were compared between controls (blue circles) and PFP-patients (orange circles). Numbers in panel A indicate percentage of reactive (CD69^+^IFNγ^+^) among total CD4 cells and CTLA-4 expression (MFI) as well as percentages of CD27- or Ki67-positive CD4 T cells after stimulation with VZV-lysate. Bars in panel B-D represent median values. To ensure robust statistics, analysis in panel C-E was restricted to samples with at least 20 antigen-specific CD4 T cells. CMV, cytomegalovirus, CTLA-4, cytotoxic T-lymphocyte antigen 4; HSV, herpes-simplex viruses; IFN, interferon; MFI, median fluorescence intensity; PFP, peripheral facial palsy; SEB, *Staphylococcus aureus* enterotoxin B; VZV, varicella-zoster virus

To analyze pathogen-specific cellular immunity in more detail, T cells were phenotypically and functionally characterized based on expression of CTLA-4, CD27 and Ki67 (Fig. 2A). The analyses shown in Fig. 2 were restricted to all samples with detectable pathogen-specific T cells. The percentage of specific T cells did not differ between the two groups (Fig. 2B). Moreover, median expression levels of the inhibitory receptor CTLA-4 were generally low (median fluorescence intensity (MFI) < 1000), despite high interindividual variabilities for VZV-, HSV- and CMV-specific T cells. Interestingly, although there were no significant differences between controls and PFP-patients, VZV-specific T cells of 9 patients showed clearly higher CTLA-4-expression than remaining patients and controls (Fig. 2C). Of note, because the number of borrelia-specific T cells was low in almost all patients and controls, further analysis of characteristic functional and phenotypical properties of borrelia-specific T cells was only possible for five controls and three patient samples, of which one patient also showed a particularly high CTLA-4-expression (Fig. 2C). Expression levels of CD27, a marker for T cell differentiation, did not differ between controls and patients. However, while VZV-, HSV- and SEB-reactive T cells were mainly CD27-positive, CD27-expression on CMV-specific T cells was low (Fig. 2D), which is a known characteristic of CMV-specific T cells [26]. The intranuclear expression of the proliferation marker Ki67 was generally low in pathogen-specific T cells of controls and patients. However, 8 out of 35 patients showed an increased percentage of Ki67-positive VZV-specific CD4 T cells (Fig. 2E). CTLA-4-, Ki67- and CD27-expression on SEB-reactive T cells did not show any significant differences between patients and controls.

### Stable levels and phenotype of pathogen-specific T cells for at least 2 weeks

**Fig. 3:**
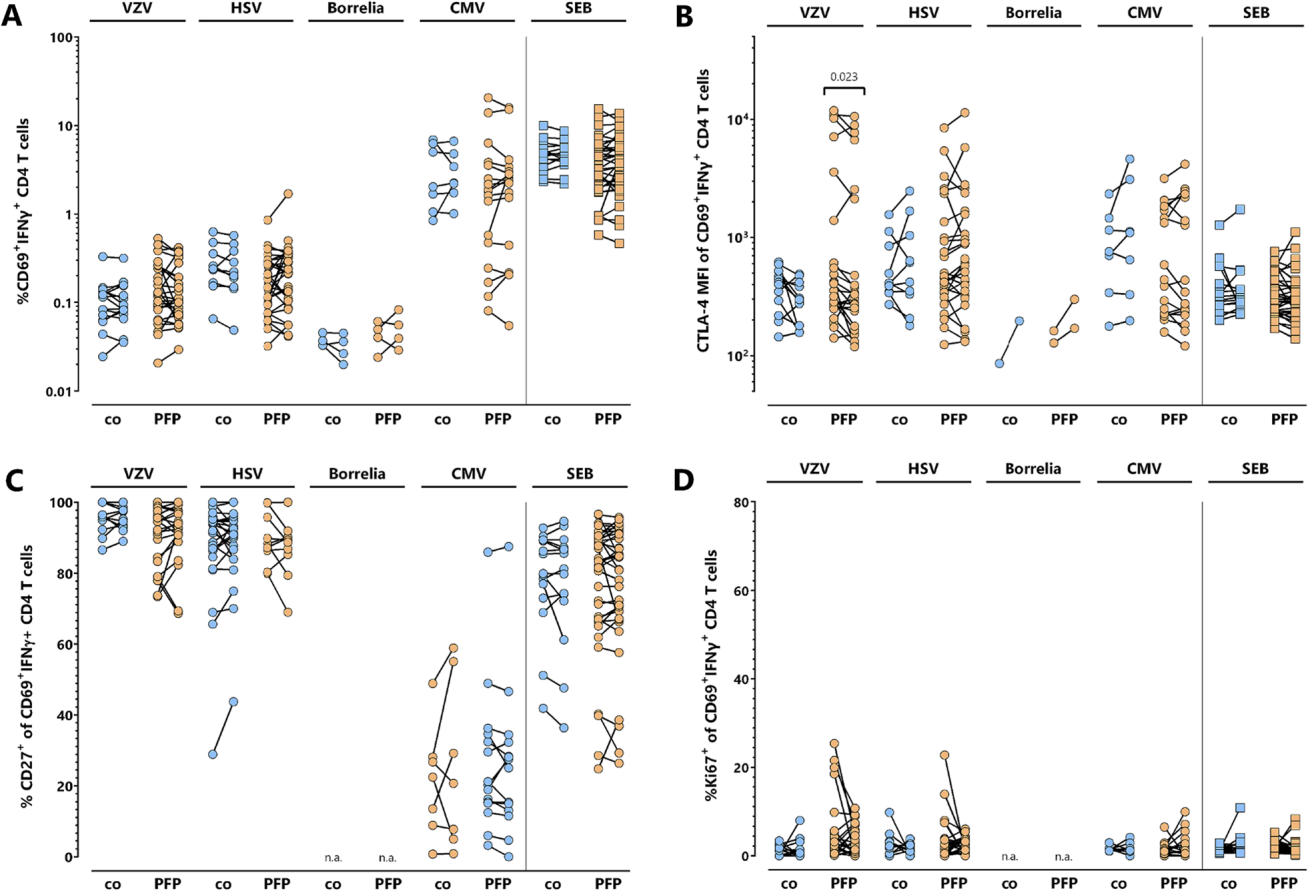
Stability of pathogen-specific T cell characteristics within 14 days after clinical presentation. Pathogen-specific T cell responses in 36 PFP-patients (orange circles) were analyzed at the beginning of symptoms and two weeks thereafter. In parallel, blood samples of 15 controls (blue circles) were additionally analyzed two weeks after the first sample recruitment. **A** Percentages of pathogen-specific and SEB-reactive CD4 T cells above detection limit were compared at both time points. **B** CTLA-4 expression as well as **C** CD27- and **D** Ki67-positivity of pathogen-specific T cells was compared at onset of symptoms. To ensure robust statistics, analysis of samples with positive pathogen-specific T cell frequencies in panel B, C and D was restricted to samples with at least 20 antigen-specific CD4 T cells. CMV, cytomegalovirus, HSV, herpes-simplex viruses; IFN, interferon; PFP; peripheral facial palsy; VZV, varicella-zoster virus

To assess potential dynamics in the quantity and phenotypical properties of the pathogen-specific T cells early after the onset of PFP, the percentage and CTLA-4-, Ki67- and CD27-expression of pathogen-specific T cells of 36 PFP-patients was analyzed at first clinical presentation and 14 days thereafter (Fig. 3). Paired samples of 15 healthy individuals served as controls, where no major changes in frequencies and expression patterns were expected. At the time of the second analysis, the median House–Brackmann grade had not yet changed significantly (*p* = 0.107). Hence, there were no significant differences in the pathogen-specific T cell properties within this time frame, except for a decrease in CTLA-4-expression on VZV-specific CD4 T cells of PFP-patients. Although this decrease was significant (*p* = 0.023), samples with high CTLA-4 expression at PFP-onset still had considerably high CTLA-4 expression levels two weeks later. Interestingly, samples of four patients with elevated percentages of Ki67^+^ VZV-specific CD4 T cells at PFP-onset showed a clear reduction of Ki67-positivity over time. Likewise, two other PFP-patients showed a similarly marked decrease in Ki67-positivity on HSV-specific T cells.

### High CTLA-4- and Ki67-expression of VZV-specific T cells as biomarker for VZV-related peripheral facial palsy

**Fig. 4:**
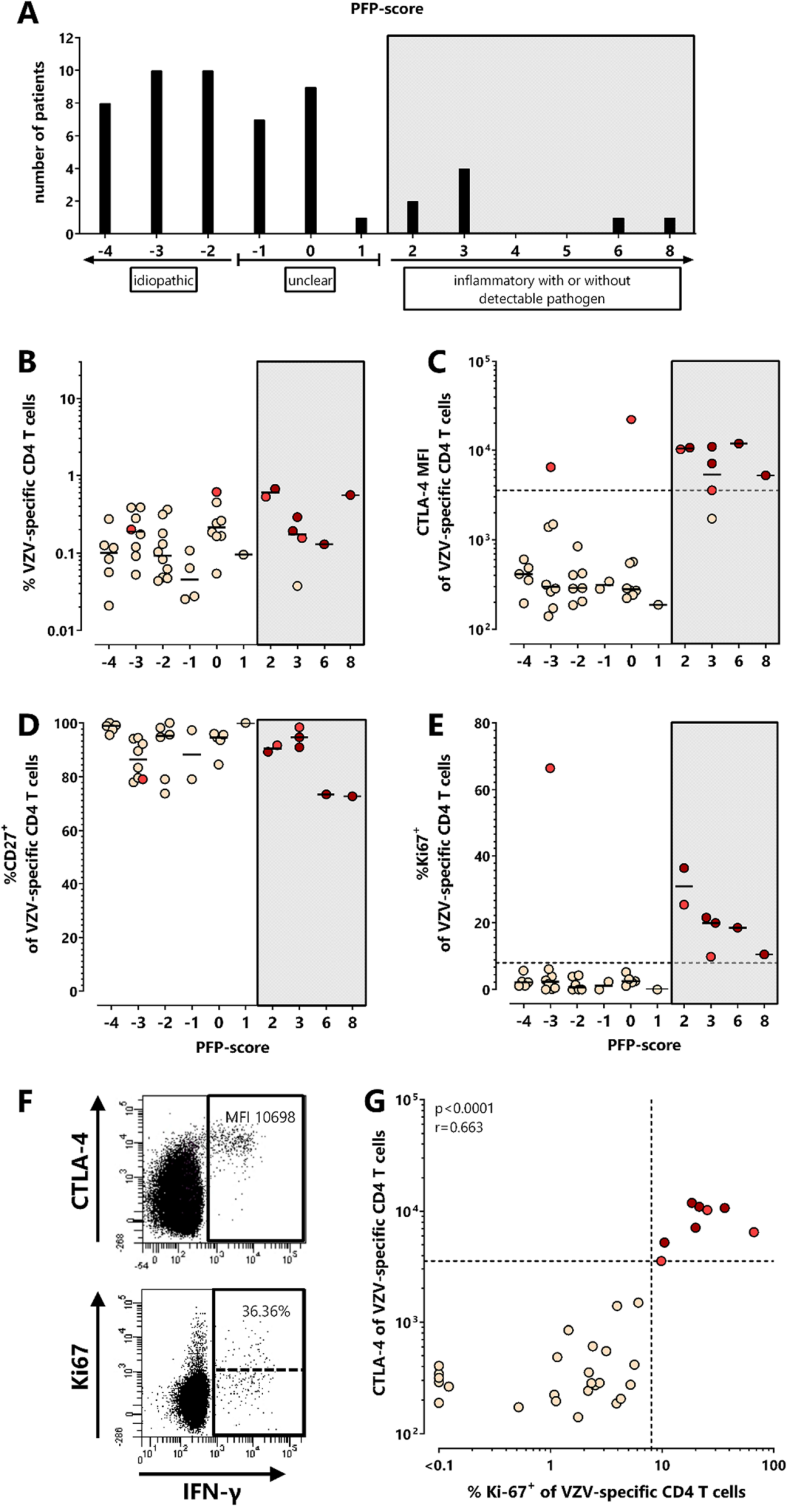
Characteristic alterations in CTLA-4 and Ki67-expression of VZV-specific T cells in patients with VZV-associated PFP. **A** Patients with PFP were subclassified according to a composite PFP-score based on the results of available routine clinical diagnostics (see Additional file 1: Table S1, *n = *53, 2 patients with Guillain-Barré syndrome were not included) to allow determination of the most probable cause of PFP. Scores < − 1 were scored as idiopathic (*n = *28), scores between ≥ − 1 and ≤ 1 as unclear (*n = *17), and scores > 1 as inflammatory with or without detectable pathogen (*n = *8, including 5 PFP-patients with VZV-induced skin disease (3 × zoster oticus, 1 × Ramsay Hunt zoster, 1 × with concomitant cervical (C2) zoster efflorescence)). **B** VZV-specific CD4 T cell levels of PFP-patients with positive VZV T cell status (*n* = 45) were compared depending on the PFP-score. Expression of **C** CTLA-4 (*n* = 39), **D** CD27 (*n* = 34), and **E** Ki67-positivity (*n* = 34) of VZV-specific T cells is shown in patients with different PFP-scores. **F** Dot plots of a 79-year-old PFP-patient with VZV-reactivation show characteristic expression of CTLA-4 (upper) and Ki67 (lower) on (IFNγ +) CD4 T cells after in vitro stimulation with VZV-antigen. **G** CTLA-4 and Ki67-expression of VZV-specific CD4 T cells was correlated among all VZV T cell-positive samples of PFP-patients where both markers were available (*n = *34). To ensure robust statistics, analysis in **C**, **D**, **E** and **G** was restricted to samples with at least 20 antigen-specific CD4 T cells. Dotted lines in **C**, **E** and **G** represent cut-offs (3579 MFI for CTLA-4 [22], and 8% for %Ki67-positive) which discriminated best between VZV-associated PFPs and non VZV-associated PFPs. Patients with dark red and light red symbols refer to patients with high CTLA-4 expression levels on VZV-specific T cells and/or high percentage of Ki67-positive VZV-specific T cells. Among them, dark red symbols refer to patients with VZV-related skin disease (3 patients with zoster oticus, 1 patient with Ramsay Hunt zoster and 1 patient with concomitant cervical (C2) zoster efflorescence). One patient (light red) did not have sufficient blood for CD27- and Ki67-staining. Numbers in panel F indicate CTLA-4-MFI and percentage of Ki67-positive of VZV-reactive CD4 T cells. CTLA-4, cytotoxic T-lymphocyte antigen 4; IFN, interferon; MFI, median fluorescence intensity; PFP, peripheral facial palsy; VZV, varicella-zoster virus

Based on available clinical parameters, two patients were diagnosed with Guillain–Barré syndrome. For the remaining 53 patients, a composite score was developed to classify PFP according to likelihood of an inflammatory or idiopathic cause, or as unclear. This score included clinical data and routine diagnostic results (Additional file 1: Table S1). Based on this score, 8 out of 53 PFP-patients showed predominant signs of an inflammatory cause with or without detectable pathogen, whereas 28 PFP-patients were classified as idiopathic. The diagnosis of PFP was classified as unclear in 17 patients (Fig. 4A, and Additional file 1: Table S2).

Among the 8 patients with inflammatory PFP-score, 5 had VZV-related skin disease with a score ≥ 2 (3 patients with zoster oticus, 1 patient with Ramsay Hunt zoster and 1 patient with concomitant cervical (C2) zoster efflorescence). All patients with detectable VZV-specific T cells are shown in Fig. 4B–G. While the percentage and CD27-expression of VZV-specific CD4 T cells of the 5 patients with zoster manifestations (labeled in dark red) did not differ from those of patients with lower PFP-scores (Fig. 4B and D), CTLA-4- and Ki67-expression were considerably higher (Fig. 4C and E), with typical dot plots shown in Fig. 4F. Their CTLA-4-MFI was above the cut-off previously established for active VZV-infection [22]. Interestingly, a similar VZV-specific CD4 T cell phenotype (CTLA-4-MFI > 3579 and > 8% Ki67-expressing cells) was also found in four more PFP-patients (labeled in light red), although the causative pathogen was either not detectable (*n = *2, score 2 and 3), standard clinical data were unavailable (*n = *1, score 0) or PFP in one pregnant woman was even classified as “idiopathic” (*n = *1, score −3). Among those, VZV-DNA or antibodies were only tested in 2 out of 4 patients. While VZV-DNA was not detectable, these two patients (with score 2 and 3) had an intermediate or positive VZV-ASI. Overall, expression levels of CTLA-4 and Ki67 on VZV-specific T cells showed a significant correlation (*r* = 0.663, *p* < 0.0001, Fig. 4G). Of note, in patients with altered phenotype of VZV-specific T cells, the properties of HSV-, CMV- and SEB-reactive T cells were unaltered and similar as in patients with normal VZV-specific T cell profile (Additional file 1: Fig. S1), which emphasizes that alterations in phenotype were VZV-specific and did not affect effector T cells in general.

### Altered phenotype of borrelia-specific T cells in a case with neuroborreliosis

In general, frequencies of borrelia-specific T cells were low or undetectable in both seronegative and seropositive individuals, and did not show any evidence for phenotypical alterations. An exception was a 9-year-old boy with acute PFP and recent history of untreated erythema migrans (1 month before). His PFP was confirmed as neuroborreliosis by a pleocytosis of 28 cells/µl in CSF and a borrelia-specific ASI of 11.07. He had 0.04% of borrelia-specific T cells. Interestingly, despite this low percentage, these cells showed particularly high CTLA-4- and Ki67-expression, whereas SEB-reactive T cell properties were unaltered except for a slightly elevated CTLA-4 expression (Additional file 1: Fig. S2). Likewise, VZV- and CMV-specific T cells had a normal phenotype and HSV-specific T cells were below detection limit. Of note, this was the only patient with both positive borrelia-specific IgG and IgM (Table 2).

## Discussion

In this study, we performed a detailed quantitative and functional analysis of immune responses against pathogens which are associated with peripheral facial palsy [5–8]. In general, we found high concordance of virus-specific antibody and T cell responses in our study cohort. While most tested parameters did not differ between patients and controls, patients had a significantly higher HSV-seroprevalence, which may support a causative role of HSV in the etiology of PFP. Nevertheless, HSV-specific T cell levels or their phenotypical characteristics did not differ in patients and controls. In contrast, we found a distinct fraction of PFP-patients with a strong expression of CTLA-4 and Ki67 on their VZV-specific T cells. This immunological alteration may hold promise as a biomarker for a causal role of VZV among individuals with PFP of unknown cause.

We have previously shown that CTLA-4 expression is specifically upregulated on VZV-specific T cells in individuals with active herpes zoster [21] or patients with VZV-related diseases of the central nervous system such as meningitis or encephalitis with a 100% sensitivity and 100% specificity for individuals with CTLA-4 MFI above the threshold of 3579 [22]. This previous study also identified 3 more patients with high CTLA-4 expression levels with unclear CNS-diagnosis, but a diagnosis of PFP [22]. The PFP-patients in the present study were heterogeneous regarding their clinical diagnosis of the underlying cause of PFP. When applying the threshold previously established for patients with acute VZV-induced CNS-disease [22], we identified 9 patients where CTLA-4 expression of VZV-specific CD4 T cells was strongly increased. This phenotype was specific for VZV-specific T cells, as characteristics of T cells with specificity towards other pathogens were similar as in controls. Among these patients, five had acute symptomatic VZV-reactivation with typical cutaneous manifestations and two additional patients showed elevated VZV-ASI, suggesting VZV-reactivation (zoster sine herpete). In contrast, a potential VZV-involvement or alternative diagnosis remained unclear in the other 2 patients based on clinical anamneses and standard laboratory diagnostics. It is therefore tempting to speculate whether these alterations may indicate a potential involvement of VZV, which may be tested in future studies by empirical treatment with appropriate anti-viral therapeutics. Notably, a large fraction of VZV-specific T cells of these patients was also Ki67-positive (9.8–66.4%), indicating recent proliferation. Based on similar observations in patients with active tuberculosis who showed higher percentages of Ki67-positive cells among *Mycobacterium tuberculosis-*specific T cells than latently infected individuals [27], increased percentages of Ki67-positive VZV-specific T cells may also argue for a causative role of VZV.

Follow-up data two weeks after the first sample acquisition revealed that the percentage of VZV-specific CD4 T cells and their CTLA-4- and CD27-expression remained almost stable or were only slightly altered. In contrast, Ki67-protein seems to be degraded during this time frame as indicated by four patients with increased percentage of Ki67-positive VZV-specific cells at time of PFP-symptoms and lower frequencies 14 days thereafter. As PFP-patients with increased CTLA-4 expression of VZV-specific T cells had a similar cellular phenotype two weeks later, this biomarker may be useful to identify VZV-associated PFPs during a diagnostic time frame of up to 14 days after onset of symptoms. Despite the fact that we have not performed any analyses at later time points, we have previously shown that characteristic phenotypical and functional alterations of VZV-specific CD4 T cells in patients with acute herpes zoster reverted back to almost normal values 3 months post VZV-reactivation [21]. Thus, future studies should investigate whether VZV-specific T cell characteristics in PFP-patients will also normalize after a longer period of time.

HSV-seroprevalence in our PFP-patient group was significantly higher (90.9%) than in our control group (52%), which may indicate a potential causative role of HSV in some patients with PFP. This high seroprevalence among PFP-patients is remarkable, as HSV-1-prevalence in the general population in Germany has rather been decreasing from 82.1% in 1997–1999 to 78.4% in 2008–2011 [28]. If the analysis is restricted to PFP-patients scored as “unclear” or “idiopathic” using standard diagnostic parameters, HSV-seropositivity was even above 95% (43/45) and thus seems to be higher than in the general German population. Support for a potential HSV-involvement is given by observations from a small study showing that HSV-DNA was specifically detected in facial nerve endoneural fluid and posterior auricular muscle of 79% of patients with “idiopathic” PFP, whereas there was no HSV-DNA in VZV-triggered PFPs or controls [3]. However, based on conflicting results on the clinical benefit of aciclovir treatment of patients with Bell’s palsy, further studies are needed to further substantiate HSV as a causative virus in PFP [4, 14, 29–31]. Furthermore, apart from increased HSV-seroprevalence in our population of PFP-patients, our study did not show any characteristic alterations in the phenotype and function of HSV-specific T cells, which may be due to the fact that HSV-reactivations may rather be associated with local recruitment of specific T cells without measurable systemic changes. Thus, unlike VZV-specific T cells, HSV-specific T cell analysis in circulation is unlikely to be useful to specifically identify patients with HSV-associated PFP.

Our study is limited by the in part low number of pathogen-specific T cells, especially after borrelia-specific stimulations. Thus, the presence of sufficient numbers of pathogen-specific T cells seems critical to enable further characterization of CTLA-4- and/or Ki67-expression. However, despite the generally low percentage of borrelia-specific T cells in our cohorts, the example of a 9-year-old boy with confirmed neuroborreliosis demonstrates that borrelia-specific T cells might be detectable during active disease. Thus, although limited by data of a single case, upregulation of CTLA-4 and Ki67 on borrelia-specific T cells may also be indicative of a specific involvement of borrelia, which warrants further study in larger cohorts of patients with neuroborreliosis. As a further limitation, we used a composite PFP-score that only provides an estimate on the likelihood of either inflammatory or idiopathic PFP, and the cause of PFP remains unclear in a large fraction of patients. Therefore, a causal association of altered T cell characteristics with VZV-involvement remains speculative in the patients with unclear diagnosis. In this regard, our findings provide an interesting basis for interventional studies with larger sample size.

In conclusion, although the quantity and phenotype of VZV-specific T cell responses did not generally differ between PFP-patients and healthy controls, the distinct increase in CTLA-4- and Ki67-expression of VZV-specific cells allows for identification of VZV-related PFP. This may even extend to patients where conventional clinical and laboratory diagnosis is unclear or limited. Combined with first evidence from a patient with neuroborreliosis, analysis of pathogen-specific T cell properties may have potential to identify VZV or borrelia as infectious causes in PFP, whereas HSV-specific T cells do not seem to be useful to identify involvement of HSV. However, the high HSV-seroprevalence in patients with idiopathic PFP is a hint for an underestimated role of HSV during development of PFP. Our findings need to be confirmed by larger sample size, and an interventional study may further define whether an altered phenotype of pathogen-specific T cells could be applied as an adjunct tool for improved diagnosis of PFP, and to guide specific anti-viral or antibiotic therapy.

### Supplementary Information

**Additional file 1: Table S1.** Clinical and diagnostic parameters for calculation of a PFP-score. **Table S2.** Individual demographic and clinical data, as well as data that were compiled into the PFP score. **Figure S1.** Distribution of the percentage and phenotype of HSV-, CMV- and SEB-reactive CD4 T cells according to PFP-score. PFP-patients (n=53) were subclassified according to PFP-score (see table S1 and figure 4A). Scores <-1 were scored as idiopathic, scores between ≥-1 and ≤1 as unclear, and scores >1 as inflammatory with or without detectable pathogen. **A** HSV-, CMV- and SEB-reactive CD4 T cell levels of T cell positive individuals (n=49 HSV T cell positive, n=27 CMV T cell positive) were compared between PFP-patients with different PFP-score. In addition, CTLA-4 (**B**), CD27- (**C**) and Ki67-expression (**D**) of reactive T cells was analyzed with regard to PFP-score. To ensure robust statistics, analysis in B, C and D was restricted to samples with at least 20 antigen-specific CD4 T cells. Lines represent median values. Patients with dark red and light red symbols refer to patients with high CTLA-4 expression levels on VZV-specific T cells and/or high percentage of Ki67-positive VZV-specific T cells (see figure 4). Among them, dark red symbols refer to patients with VZV-related skin disease (3 patients with zoster oticus, 1 patient with Ramsay Hunt zoster and 1 patient with concomitant cervical (C2) zoster efflorescence). CMV, cytomegalovirus; CTLA-4, cytotoxic T-lymphocyte antigen 4; IFN, interferon; HSV, herpes-simplex viruses; MFI, median fluorescence intensity; PFP, peripheral facial palsy; SEB, *Staphylococcus aureus* enterotoxin B. **Figure S2.** Distinct changes of borrelia-specific T cell properties in a case of neuroborreliosis-related PFP. Borrelia-specific (**A**) and SEB-reactive (**B**). CD4 T cells of a 9-year-old boy with acute PFP and confirmed neuroborreliosis were determined after antigen-specific stimulation and flow cytometric detection. Numbers in each dot plot indicate percentages of reactive (CD69^+^IFNγ^+^) CD4 T cells (upper panels), CTLA-4 (middle panels) and Ki67-expression of reactive CD4 T cells (lower panels), respectively. Follow-up data of this patient were not available. CTLA-4, cytotoxic T-lymphocyte antigen 4; IFN, interferon; MFI, median fluorescence intensity; PFP, peripheral facial palsy; SEB, *Staphylococcus aureus* enterotoxin B.

## Data Availability

The data that support the findings of this study are available from the corresponding author upon request.

## Abbreviations

ASI: Antibody specific index
CMV: Cytomegalovirus
CTLA-4: Cytotoxic T-lymphocyte antigen-4
HSV: Herpes-simplex virus
IFN: Interferon
MFI: Median fluorescence intensity
PFP: Peripheral facial palsy
SEB: Staphylococcus aureus enterotoxin B
VZV: Varicella-zoster virus
