# Supplementary material for: Alterations in pathogen-specific cellular and humoral immunity associated with acute peripheral facial palsy of infectious origin
Source: J Neuroinflammation. 2023 Oct 25;20:246. doi: 10.1186/s12974-023-02933-4 (PMC10598953; doi:10.1186/s12974-023-02933-4)
Supplement: Supplementary file 1 — Additional file 1: Table S1. Clinical and diagnostic parameters for calculation of a PFP-score. Table S2. Individual demographic and clinical data, as well as data that were compiled into the PFP score. Figure S1. Distribution of the percentage and phenotype of HSV-, CMV- and SEB-reactive CD4 T cells according to PFP-score. PFP-patients (n=53) were subclassified according to PFP-score (see table S1 and figure 4A). Scores <-1 were scored as idiopathic, scores between ≥-1 and ≤1 as unclear, and scores >1 as inflammatory with or without detectable pathogen. A HSV-, CMV- and SEB-reactive CD4 T cell levels of T cell positive individuals (n=49 HSV T cell positive, n=27 CMV T cell positive) were compared between PFP-patients with different PFP-score. In addition, CTLA-4 (B), CD27- (C) and Ki67-expression (D) of reactive T cells was analyzed with regard to PFP-score. To ensure robust statistics, analysis in B, C and D was restricted to samples with at least 20 antigen-specific CD4 T cells. Lines represent median values. Patients with dark red and light red symbols refer to patients with high CTLA-4 expression levels on VZV-specific T cells and/or high percentage of Ki67-positive VZV-specific T cells (see figure 4). Among them, dark red symbols refer to patients with VZV-related skin disease (3 patients with zoster oticus, 1 patient with Ramsay Hunt zoster and 1 patient with concomitant cervical (C2) zoster efflorescence). CMV, cytomegalovirus; CTLA-4, cytotoxic T-lymphocyte antigen 4; IFN, interferon; HSV, herpes-simplex viruses; MFI, median fluorescence intensity; PFP, peripheral facial palsy; SEB, Staphylococcus aureus enterotoxin B. Figure S2. Distinct changes of borrelia-specific T cell properties in a case of neuroborreliosis-related PFP. Borrelia-specific (A) and SEB-reactive (B). CD4 T cells of a 9-year-old boy with acute PFP and confirmed neuroborreliosis were determined after antigen-specific stimulation and flow cytometric detection. Numbers in each dot plot indic [file 12974_2023_2933_MOESM1_ESM.docx]

# Additional files

# Alterations in pathogen-specific cellular and humoral immunity associated with acute peripheral facial palsy of infectious origin

Leyla Mohammad^1,*^; Mathias Fousse^2,*^, MD; Gentiana Wenzel^3^, MD; Marina Flotats Bastardas^4^, MD; Klaus Faßbender^2^, MD; Ulrich Dillmann^2^, MD; Bernhard Schick^3^, Michael Zemlin^4^, MD; Barbara C. Gärtner^5^, MD; Urban Sester^6^, MD; David Schub^1^, PhD; Tina Schmidt^1^, PhD; and Martina Sester^1^, PhD

The supplement contains a description of a composite PFP score, two supplementary table S1 and S2, and two supplementary figures S1 and S2.

# Additional Information

## PFP-score as composite estimate for assignment of the most probable cause of PFP

PFP-patients were subclassified according to clinical data and results of available routine diagnostics. Information on five parameters were scored individually and were compiled in a composite PFP-score as detailed in table S1 to allow assignment of the most probable cause of PFP. The PFP-score includes individual scoring of (1) pathogen detection from cerebrospinal fluid (CSF), (2) cell numbers in CSF, (3) early electrophysiological testing, (4) clinical response to anti-infective treatment or steroids, and (5) infectious clinical stigmata (erythema migrans or herpes zoster). A positive individual score was chosen for parameters supporting an infection-related inflammation, whereas a negative score was chosen for parameters supporting an idiopathic etiology. Total PFP-scores <-1 were classified as idiopathic, scores between ≥-1 and ≤1 as unclear, and scores >1 as inflammatory with or without detectable infectious pathogen.

Individual patient clinical data and demographic information, as well as individual results on parameters included in the score are summarized in table S2.

# Additional Tables

## Table S1: Clinical and diagnostic parameters for calculation of a PFP-score

| **PFP-score^#^ individual scoring** | |
| --- | --- |
| **(1)** Detection of pathogen^$^ in CSF by PCR or microscopy  yes  no or n.d. | +3  0 |
| **(2)** Cell number in CSF  >5 cells/µl (pleocytosis)  5 cells/µl (borderline)  <5 cells/µl (normal)  n.d. | +2  0  -2  0 |
| **(3)** Early electrophysiological testing*  Lesion outside the facial nerve canal (no canalis facialis)  Lesion inside the facial nerve canal (canalis facialis)  incomplete or not reliable or n.d. | +1  -1  0 |
| **(4)** Response to treatment  improvement after anti-infective treatment (anti-viral, anti-bacterial agents)  worsening after steroids  improvement/worsening after triple therapy (steroids + anti-viral + anti-bacterial)  improvement after steroids | +1  +1  0  -1 |
| **(5)** Stigma: visible herpes zoster or erythema migrans | +1 |

^#^PFP-patients with Guillain-Barré syndrome (n=2) were excluded from analysis with PFP-score; ^$^VZV and HSV was tested by PCR, and bacteria were tested by microscopy primarily in patients with CSF cell counts ≥5/µl; *performed within 4 days after onset of disease; n.d. not determined; total PFP-scores of <-1 were scored as idiopathic, scores between ≥-1 and ≤1 as unclear, and scores >1 as inflammatory with or without detectable infectious pathogen.

## Table S2: Individual demographic and clinical data, as well as data that were compiled into the PFP score.

|  | **Demographics and clinical data** | | | | | **Data compiled in the PFP score** | | | | | | | | | | | |
| --- | --- | --- | --- | --- | --- | --- | --- | --- | --- | --- | --- | --- | --- | --- | --- | --- | --- |
| **ID** | **Sex** | **age** | **PFP side** | **HB-grading** | **Clinical comments** | **(1)**  **Pathogen detection (PCR or microscopy)** | **(1)**  **Score** | **(2)**  **CSF cells/µl** | **(2)**  **Score** | **(3)**  **Early electrophysiological testing** | **(3)**  **Score** | **treatment** | **(4)**  **Response to treatment** | **(4)**  **Score** | **(5)**  **Stigma** | **(5)**  **Score** | **(1-5)**  **Total**  **PFP Score** |
| 19888 | female | 65 | left | V |  | n.d. | 0 | 1 | -2 | canalis facialis | -1 | steroids | improvement | -1 | no | 0 | **-4** |
| 19972 | male | 13 | left | III |  | n.d. | 0 | 3 | -2 | canalis facialis | -1 | steroids | improvement | -1 | no | 0 | **-4** |
| 20038 | male | 48 | right | V |  | n.d. | 0 | 4 | -2 | canalis facialis | -1 | steroids | improvement | -1 | no | 0 | **-4** |
| 20069 | female | 42 | right | III |  | n.d. | 0 | 2 | -2 | canalis facialis | -1 | steroids | improvement | -1 | no | 0 | **-4** |
| 20324 | female | 60 | left | III |  | n.d. | 0 | 1 | -2 | canalis facialis | -1 | steroids | improvement | -1 | no | 0 | **-4** |
| 20362 | male | 19 | left | IV |  | n.d. | 0 | 0 | -2 | canalis facialis | -1 | steroids | improvement | -1 | no | 0 | **-4** |
| 20532 | female | 28 | left | III-IV |  | n.d. | 0 | 3 | -2 | canalis facialis | -1 | steroids | improvement | -1 | no | 0 | **-4** |
| 21391 | female | 36 | right | IV |  | n.d. | 0 | 2 | -2 | canalis facialis | -1 | steroids | improvement | -1 | no | 0 | **-4** |
| 19344 | male | 55 | right | II-III |  | n.d. | 0 | 1 | -2 | incomplete | 0 | steroids | improvement | -1 | no | 0 | **-3** |
| 19359 | male | 22 | right | II |  | n.d. | 0 | 4 | -2 | n.d. | 0 | steroids | improvement | -1 | no | 0 | **-3** |
| 19499 | male | 80 | left | III |  | n.d. | 0 | 0 | -2 | n.d. | 0 | steroids | improvement | -1 | no | 0 | **-3** |
| 19781 | female | 13 | left |  |  | n.d. | 0 | 2 | -2 | n.d. | 0 | steroids | improvement | -1 | no | 0 | **-3** |
| 20055 | male | 58 | right | IV |  | n.d. | 0 | 3 | -2 | n.d. | 0 | steroids | improvement | -1 | no | 0 | **-3** |
| 20715 | female | 56 | left | III-IV |  | n.d. | 0 | 1 | -2 | canalis facialis | -1 | steroids | unchanged | 0 | no | 0 | **-3** |
| 20796 | male | 45 | left | n.d. |  | n.d. | 0 | 3 | -2 | canalis facialis | -1 | steroids | unclear | 0 | no | 0 | **-3** |
| 21175 | female | 34 | right | II | 39th week pregnancy | n.d. | 0 | 3 | -2 | canalis facialis | -1 | steroids | unchanged | 0 | no | 0 | **-3** |
| 21489 | male | 55 | left | V |  | n.d. | 0 | 2 | -2 | canalis facialis | -1 | steroids | unclear | 0 | no | 0 | **-3** |
| 21715 | male | 20 | left | IV |  | n.d. | 0 | 2 | -2 | n.d. | 0 | steroids | improvement | -1 | no | 0 | **-3** |
| 19486 | male | 51 | left | III-IV |  | n.d. | 0 | n.d. | 0 | canalis facialis | -1 | steroids | improvement | -1 | no | 0 | **-2** |
| 19529 | male | 61 | left | V |  | n.d. | 0 | 2 | -2 | incomplete | 0 | steroids | unchanged | 0 | no | 0 | **-2** |
| 20044 | male | 43 | right | IV |  | n.d. | 0 | 0 | -2 | n.d. | 0 | triple therapy | unclear | 0 | no | 0 | **-2** |
| 20045 | male | 15 | right | III | PFP left four years ago | n.d. | 0 | 3 | -2 | n.d. | 0 | triple therapy | improvement | 0 | no | 0 | **-2** |
| 20275 | male | 28 | right | IV |  | n.d. | 0 | 2 | -2 | n.d. | 0 | steroids | unclear | 0 | no | 0 | **-2** |
| 20465 | female | 25 | right | IV |  | n.d. | 0 | 0 | -2 | n.d. | 0 | steroids | unclear | 0 | no | 0 | **-2** |
| 21072 | female | 50 | right | V |  | n.d. | 0 | 1 | -2 | canalis facialis | -1 | steroids | worsening | 1 | no | 0 | **-2** |
| 21343 | male | 65 | right | V |  | n.d. | 0 | 3 | -2 | canalis facialis | -1 | steroids | worsening | 1 | no | 0 | **-2** |
| 21575 | male | 49 | left | IV |  | n.d. | 0 | 3 | -2 | no canalis facialis | 1 | steroids | improvement | -1 | no | 0 | **-2** |
| 21656 | female | 48 | right | II |  | n.d. | 0 | 1 | -2 | no canalis facialis | 1 | steroids | improvement | -1 | no | 0 | **-2** |
| 19364 | female | 64 | left | II-III |  | n.d. | 0 | n.d. | 0 | canalis facialis | -1 | no specific treatment | improvement | 0 | no | 0 | **-1** |
| 19639 | male | 78 | left | IV | PFP left eight years ago | n.d. | 0 | n.d. | 0 | canalis facialis | -1 | steroids | unclear | 0 | no | 0 | **-1** |
| 19836 | male | 26 | right | IV |  | negative | 0 | 5 | 0 | canalis facialis | -1 | triple therapy | worsening | 0 | no | 0 | **-1** |
| 20377 | female | 20 | right | V |  | n.d. | 0 | 1 | -2 | no canalis facialis | 1 | steroids | unclear | 0 | no | 0 | **-1** |
| 21036 | male | 58 | left | IV |  | n.d. | 0 | 4 | -2 | no canalis facialis | 1 | triple therapy | improvement | 0 | no | 0 | **-1** |
| 21275 | female | 30 | left | III |  | n.d. | 0 | 2 | -2 | no canalis facialis | 1 | steroids | unclear | 0 | no | 0 | **-1** |
| 21362 | male | 64 | left | n.d. |  | n.d. | 0 | 1 | -2 | n.d. | 0 | steroids | worsening | 1 | no | 0 | **-1** |
| 19338 | female | 23 | left | I-II |  | negative | 0 | 5 | 0 | incomplete | 0 | antiviral/antibacterial | unclear | 0 | no | 0 | **0** |
| 19356 | female | 36 | left | II-III |  | n.d. | 0 | n.d. | 0 | incomplete | 0 | no specific treatment | unchanged | 0 | no | 0 | **0** |
| 19530 | male | 40 | left | IV-V |  | negative | 0 | 5 | 0 | n.d. | 0 | steroids | unclear | 0 | no | 0 | **0** |
| 19602 | male | 72 | right | II | PFP left two years ago | n.d. | 0 | n.d. | 0 | no reliable assignment | 0 | no specific treatment | unchanged | 0 | no | 0 | **0** |
| 19671 | female | 65 | left | IV |  | n.d. | 0 | n.d. | 0 | n.d. | 0 | triple therapy | unclear | 0 | no | 0 | **0** |
| 19778 | male | 56 | left | II |  | negative | 0 | 5 | 0 | n.d. | 0 | antiviral/antibacterial | unchanged | 0 | no | 0 | **0** |
| 19782 | male | 30 | right | III |  | n.d. | 0 | n.d. | 0 | n.d. | 0 | triple therapy | unclear | 0 | no | 0 | **0** |
| 20554 | male | 75 | left | II |  | n.d. | 0 | n.d. | 0 | n.d. | 0 | antiviral/antibacterial | unclear | 0 | no | 0 | **0** |
| 21230 | male | 56 | left | V |  | n.d. | 0 | 2 | -2 | no canalis facialis | 1 | steroids | worsening | 1 | no | 0 | **0** |
| 21561 | male | 59 | left | IV-V |  | n.d. | 0 | 7 | 2 | canalis facialis | -1 | triple therapy | worsening | 0 | no | 0 | **1** |
| 20225 | female | 79 | left | II-III |  | negative | 0 | 2 | 0^§^ | no canalis facialis | 1 | antiviral/steroids | improvement | 0^$^ | Herpes Zoster C2^&^ | 1 | **2** |
| 21914 | male | 32 | left | IV |  | negative | 0 | 184 | 2 | canalis facialis | -1 | antiviral/antibacterial | improvement | 1 | no | 0 | **2** |
| 20175 | female | 50 | right | III-IV |  | negative | 0 | 17 | 2 | no reliable assignment | 0 | antiviral/antibacterial | improvement | 1 | no | 0 | **3** |
| 20343 | male | 59 | right | III | Ramsay-Hunt syndrome | negative | 0 | 11 | 2 | incomplete | 0 | triple therapy | worsening | 0 | Herpes Zoster oticus + V2, V3* | 1 | **3** |
| 21033 | male | 9 | right | n.d. | history of untreated Erythema migrans 1 month earlier | negative | 0 | 28 | 2 | n.d. | 0 | antibacterial | improvement | 1 | no | 0 | **3** |
| 22180 | male | 40 | left | VI |  | negative | 0 | 63 | 2 | n.d. | 0 | antiviral/steroids | unchanged | 0 | Herpes Zoster oticus | 1 | **3** |
| 22250 | female | 81 | left | II-III |  | VZV-PCR positive | 3 | 142 | 2 | canalis facialis | -1 | antiviral/antibacterial | improvement | 1 | Herpes Zoster oticus | 1 | **6** |
| 20056 | female | 52 | left | III - IV |  | VZV-PCR positive | 3 | 80 | 2 | no canalis facialis | 1 | antiviral/antibacterial | improvement | 1 | Herpes Zoster oticus | 1 | **8** |
| 19806^#^ | male | 53,0 | bilateral | II - III | Guillain-Barré syndrome | n.a. | n.a. | n.a. | n.a. | n.a. | n.a. | n.a. | n.a. | n.a. | n.a. | n.a. | **n.d.** |
| 20176^#^ | male | 63,3 | bilateral | IV-V | Guillain-Barré syndrome | n.a. | n.a. | n.a. | n.a. | n.a. | n.a. | n.a. | n.a. | n.a. | n.a. | n.a. | **n.d.** |

^#^PFP-patients with Guillain-Barré syndrome (n=2) were excluded from analysis with PFP-score; ^§^cell count in CSF was scored as 0 due to a history of therapy with acyclovir and steroids for one week; ^$^response to treatment was scored as 0 as no specific evaluation was possible due to the combination of anti-infective and anti-inflammatory therapy; ^&^cervical radicular herpes zoster; *herpes zoster of the trigeminal nerve.

# Additional figures

## Figure S1


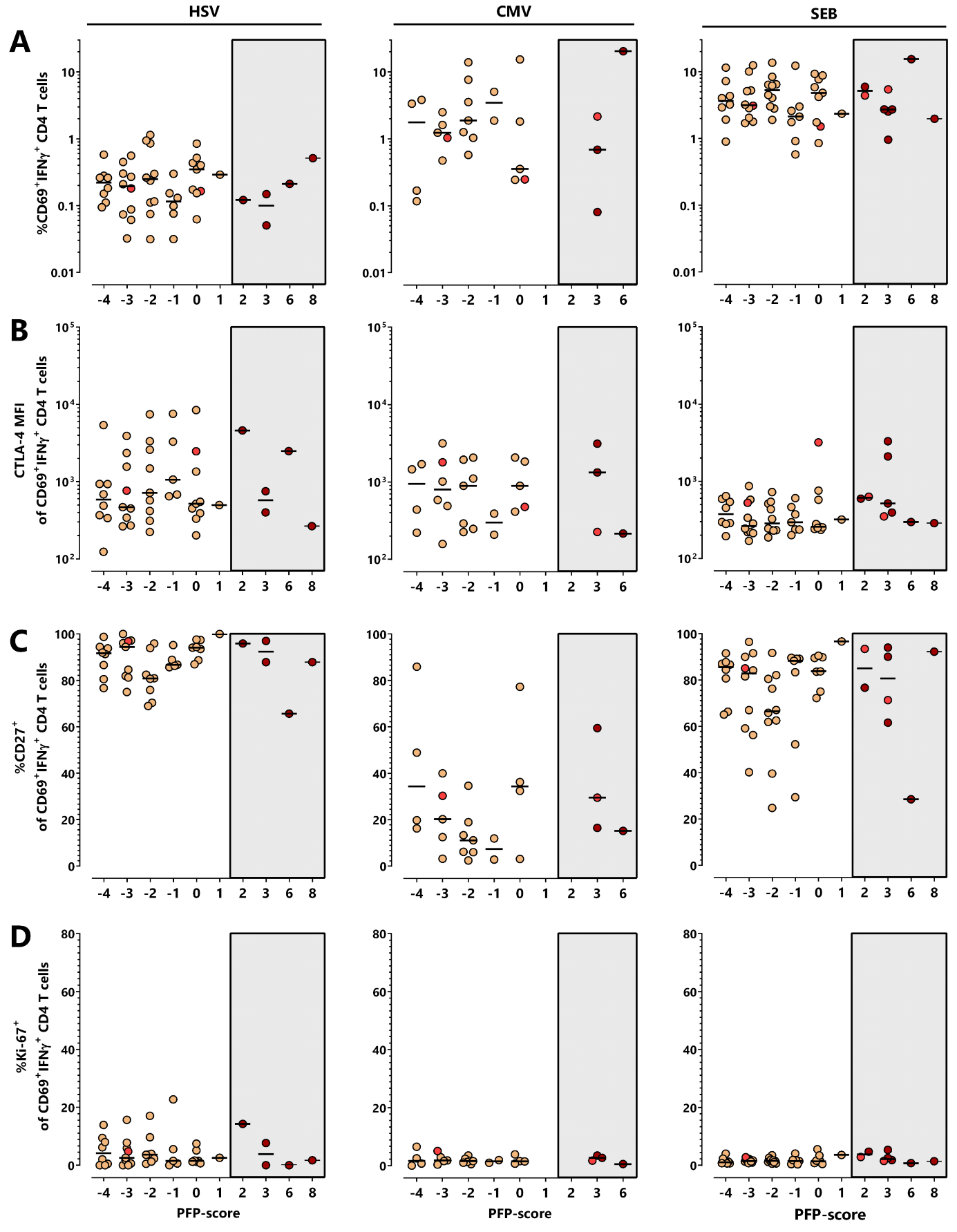


**Figure S1: Distribution of the percentage and phenotype of HSV-, CMV- and SEB-reactive CD4 T cells according to PFP-score.** PFP-patients (n=53) were subclassified according to PFP-score (see table S1 and figure 4A). Scores <-1 were scored as idiopathic, scores between ≥-1 and ≤1 as unclear, and scores >1 as inflammatory with or without detectable pathogen. **(A)** HSV-, CMV- and SEB-reactive CD4 T-cell levels of T-cell positive individuals (n=49 HSV T-cell positive, n=27 CMV T-cell positive) were compared between PFP-patients with different PFP-score. In addition, CTLA-4 **(B)**, CD27- **(C)** and Ki67-expression **(D)** of reactive T cells was analyzed with regard to PFP-score. To ensure robust statistics, analysis in B, C and D was restricted to samples with at least 20 antigen-specific CD4 T cells. Lines represent median values. Patients with dark red and light red symbols refer to patients with high CTLA-4 expression levels on VZV-specific T cells and/or high percentage of Ki67-positive VZV-specific T cells (see figure 4). Among them, dark red symbols refer to patients with VZV-related skin disease (3 patients with zoster oticus, 1 patient with Ramsay hunt zoster and 1 patient with concomitant cervical (C2) zoster efflorescence). CMV, cytomegalovirus; CTLA-4, cytotoxic T-lymphocyte antigen 4; IFN, interferon; HSV, herpes-simplex viruses; MFI, median fluorescence intensity; PFP, peripheral facial palsy; SEB, *Staphylococcus aureus* enterotoxin B.

## Figure S2


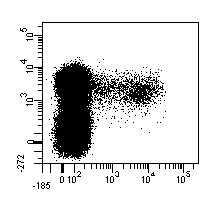

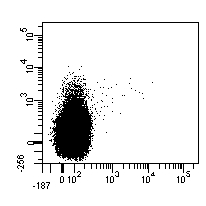

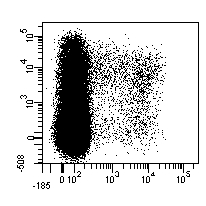

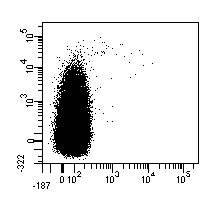

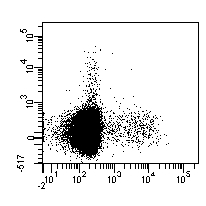

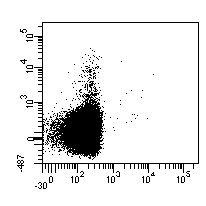


**30.51%**

**1.79%**

**MFI 26866**

**MFI 3304**

**0.04%**

**2.53%**

**Borrelia**

**SEB**

**IFNγ**

**Ki67**

**IFNγ**

**CTLA-4**

**CD69**

**Figure S2: Distinct changes of borrelia-specific T-cell properties in a case of neuroborreliosis-related PFP.** Borrelia-specific **(A)** and SEB-reactive **(B)** CD4 T cells of a 9-years old boy with acute PFP and confirmed neuroborreliosis were determined after antigen-specific stimulation and flow cytometric detection. Numbers in each dot plot indicate percentages of reactive (CD69^+^IFNγ^+^) CD4 T cells (upper panels), CTLA-4 (middle panels) and Ki67-expression of reactive CD4 T cells (lower panels), respectively. Follow-up data of this patient were not available. CTLA-4, cytotoxic T-lymphocyte antigen 4; IFN, interferon; MFI, median fluorescence intensity; PFP, peripheral facial palsy; SEB, *Staphylococcus aureus* enterotoxin B.
